# A Deeper Look at the “Neural Correlate of Consciousness”

**DOI:** 10.3389/fpsyg.2016.01044

**Published:** 2016-07-26

**Authors:** Sascha Benjamin Fink

**Affiliations:** Institute 3, Philosophy-Neuroscience-Cognition Program, Otto-von-Guericke-Universität MagdeburgMagdeburg, Germany

**Keywords:** neuroscience of consciousness, experimentum crucis, neural correlates of consciousness (NCCs), integrated information theory, recursive processing

## Abstract

A main goal of the neuroscience of consciousness is: find the neural correlate to conscious experiences (NCC). When have we achieved this goal? The answer depends on our operationalization of “NCC.” Chalmers (2000) shaped the widely accepted operationalization according to which an NCC is a neural *system* with a state which is *minimally sufficient (but not necessary)* for an experience. A deeper look at this operationalization reveals why it might be unsatisfactory: (i) it is not an operationalization of a correlate for occurring experiences, but of the *capacity* to experience; (ii) it is unhelpful for certain cases which are used to motivate a search for neural correlates of consciousness; (iii) it does not mirror the usage of “NCC” by scientists who seek for unique correlates; (iv) it hardly allows for a form of comparative testing of hypotheses, namely *experimenta crucis*. Because of these problems (i–iv), we ought to amend or improve on Chalmers's operationalization. Here, I present an alternative which avoids these problems. This “NCC2.0” also retains some benefits of Chalmers's operationalization, namely being compatible with contributions from extended, embedded, enacted, or embodied accounts (*4E*-accounts) and allowing for the possibility of non-biological or artificial experiencers.

Phenomenal consciousness is currently the target of a booming neuroscientific research program, which intends to find the neural correlates of consciousness (NCCs). “Neural correlate of consciousness” is then a core concept of this neuroscience of consciousness for at least two reasons. First, because “NCC” defines the research goal of *experiments*: find neural correlates of some conscious experiences, e.g. of the seen (rather than suppressed) image in binocular rivalry tasks, of hallucinated voices in schizophrenic episodes, of out-of-body-experiences, of red-experiences, of pains, of our dreams of flying, etc. Second, because the notion of an “NCC” determines which cases are to be considered as test instances for general scientific theories of consciousness: any scientific theory of consciousness is only satisfactory if it says something about the neural signature of consciousness, at least in us humans. In some instances (like binocular rivalry, dreaming, hallucination, etc.), neural activation may be sufficient for certain experiences, because the co-occurring events in the environment do not determine the character or content of these experiences. Thus, any general scientific theory of consciousness ought to implicate NCCs in order to be testable:^1^ if such a general theory implicates some neural activation as a plausible NCC, but that activation *fails* to correlate with conscious experience, then this gives us reason to reject that theory as a valid theory of consciousness. (At the very least, this lowers its credibility.) If such a general theory of consciousness fails to indicate *any* empirically determinable NCC, then that theory is either false, incomplete, or unsatisfactory.

NCCs are touchstones for (and implicated by) any theory of consciousness, but “NCC” is not defined *by* any of these theories. It is a notion that all theories in that field must share equally in order to be comparable.

However, it is not obvious what makes something fall under the term “NCC.” It is not a term of natural language, so we cannot claim prior understanding, familiarity, or competence with its use. And we have to defend it against triviality, because events may correlate with each other to any degree between −1 and +1, synchronously or asynchronously, and decreases can correlate with increases, absences with presences. If we do not introduce any restrictions on correlation, most events will correlate with each other—but mainly in an irrelevant fashion. If we accept any neural event correlating in any arbitrary way to an experience as an NCC, then this endeavor may become dangerously meaningless. So we have to specify which kinds of correlations are the relevant or “right” ones.^2^

Which constraints on correlation may count as relevant or “right” may depend on our pragmatic interests, i.e., what we want to do with our knowledge of NCCs. For example, if we want to know if someone is *currently* experiencing, e.g., a coma patient or a dreamer, we will prefer to know *synchronous* correlates. If we want to *prevent* experiences from arising (e.g., we want to prevent a patient from regaining consciousness during surgery), we may prefer knowing *preceding* (asynchronous) correlates (because detection of these types of correlates would leave the anesthesiologist time for interventions). I discuss other well motivated constraints later on. So how ought we to understand “NCC”? What should we want an NCC to be in order to maximize its applicability?

First, I focus on how the term “NCC” was introduced by Crick (1995). His operationalization had one major flaw, which was pointed out by Chalmers (2000). Chalmers's operationalization is widely accepted by philosophers and neuroscientists alike. Even though Chalmers's operationalization avoids some problems, it is not fully satisfactory, for which I argue in section 2: (i) it is not an operationalization of a neural correlate of experiences themselves, but of the *potential* to have such experiences; (ii) it is impractical and unhelpful for cases which are commonly used to motivate a search for NCCs; (iii) it does not mirror how “NCC” is used by scientists, who seek for *unique* correlates, and it leaves the relation between data and theory unclear; (iv) it hardly allows for *experimenta crucis*, a form of comparative testing of hypotheses widely held as important and beneficial to scientific progress. In section 3, I present an alternative operationalization and elaborate how it addresses these worries.

## 1. Operationalizing “neural correlate of consciousness”

Operationalizing is an *a priori* endeavor where one determines what it is that one searches for empirically: we introduce (or alter) a term *t* by defining *t* operationally. More specifically, we state the (preferably detectable) circumstances by which we determine whether something falls under *t* or not. If *t* has a previous use, we ought to preserve its connotations; if *t* has no previous use, we can operationalize as we please; but whether we adopt this rather than that operationalization of *t* will hinge on its usefulness: what do we gain by thinking of *t* in this way rather than another?

Although “neural correlate of consciousness” has been used before Francis Crick's *The Astonishing Hypothesis* (1995),^3^ his book is how the term got traction. Crick (1995, p. 9) writes that we might possibly “explain to you the *neural correlate* of your seeing red. In other words, we may be able to say that you perceive red if and only if certain neurons (and/or molecules) in your head behave in a certain way.” This is no straightforward operationalization, but the phrase *if and only if* suggests that Crick believed some neural activation to be *both* sufficient *and* necessary for an experience: whenever neurons *n*_1_, …, *n*_*x*_ behave in such-and-such a way, one experiences *red*—and when they are inactive, one does not experience *red*.

### 1.1. The mere-sufficiency-constraint

Chalmers (2000, p. 24ff) took issue with the requirement that the activation of some group of individual neurons ought to count as sufficient *and necessary* for an experience. There are good *a priori* and *a posteriori* reasons to reject such a “necessity-constraint.” First, if we were to accept and take some individual neural activation to be *necessary* for an experience, we reject several reasonable possibilities *a priori*. For example, we rule out *a priori* that there could be artificial experiencers, i.e., non-biotic conscious machines or programs.^4^ Second, by accepting the necessity-constraint, we rule out *a priori* that we could preserve consciousness with silicon brain prostheses: if *neural* activation is necessary, then replacing a lesion with any (however complicated) microprocessors would lead to a loss of consciousness. There is hardly any conclusive uncontested argument against these possibilities. Third, by accepting the necessity-constraint, we rule out *a priori* that the activation of *different* populations of neurons in a brain could bring about *the same* experience.^5^ Why should we rule this out *a priori*, if redundancy is *a posteriori* plausible? Tiny lesions (e.g., due to the natural death of individual neurons) do not seem to affect our ability to have certain experiences, because there are enough other neurons which still play their contributing roles. I do not notice the death of individual neurons or even columns by focusing on my experiences. Only if these lesions are large enough do they affect my phenomenal experiences in a noticeable way. Fourth, consider cases of plasticity, where patients regain the capability for certain experiences after a lesion just like they may regain certain cognitive functions (see e.g., Borgstein and Grootendorst, 2002; Stein and Hoffman, 2003; Wieloch and Nikolich, 2006; Murphy and Corbett, 2009; Wittenburg, 2010). Because we should rule out any of these possibilities *a priori*, we are lead to a “mere-sufficiency-constraint”: for any operationalization of “NCC,” any individual neural activation ought to count at most as *sufficient* for an experience, *but not as necessary*. For otherwise, neither artificial experiencers, nor silicon brain prostheses, nor redundancy or plasticity are possible.

### 1.2. The minimality-constraint

Rejecting the necessity constraint has an unwelcome consequence: most neural activations which are sufficient for an experience will also be uninformative. Say I hear Wagner's *Tristan Chord* while attending an opera at Bayreuth. The activation of my brain *as a whole* is then sufficient for e.g., an experience of the note *f*, because *f* is part of this chord. But the activation of my brain as a whole is also sufficient for experiencing *d#*, the experience of Isolde's red dress, the pain I feel from the uncomfortable chairs, and so on. Knowing that the activation of my whole brain *now* is a neural correlate of hearing *f* is uninformative, because I might never have this specific overall brain activation again in my life. According to Chalmers (2000, p. 24f, 32), what we desire to know is the *minimal* system whose activation corresponds to an experience: which minimal subset of the activation of my whole brain is by itself sufficient for hearing *f*. We may call this the “minimality-constraint”: only the smallest subset of a neural activation which *by itself* can bring about an experience ought to count as that experience's NCC. By accepting the minimality-constraint, we cannot count the whole activation of a brain as a neural correlate of an experience *e* although, taken literally, it correlates with *e* in some sense.

### 1.3. Chalmers's operationalization

Chalmers argues for both the mere-sufficiency-constraint and the minimality-constraint and incorporates them in his operationalization:

**The Chalmers-NCC:** “An NCC is a minimal neural system *N* such that there is a mapping from states of *N* to states of consciousness, where a given state of *N* is sufficient, under conditions *C*, for the corresponding state of consciousness.” (Chalmers, 2000, p. 31)^6^

Most follow Chalmers in his outline: Mormann and Koch (2007) talk about “neural mechanisms or events which are sufficient for consciousness,”^7^ and similar wording can be found in Aru et al. (2012), Bayne and Hohwy (2013), Block (2005, p. 46), Hohwy (2007, 2009), Hohwy and Frith (2004), Tononi and Koch (2008), and others. Although none of the mentioned authors quotes Chalmers (2000), the similarities are obvious. It is therefore reasonable to assume that the Chalmers-NCC is the default understanding of “NCC” in the neuroscience of consciousness.

Note that this operationalization does not explicitly specify whether it refers to types or tokens: clearly *my* brain has some subsystem with a state sufficient for my red-experience *now*. This would count as an NCC. But we clearly want a notion of NCC that transcends one individual brain. Thus, we should also allow for inter-individual *types* of correlates for inter-individual *types* of experiences: my mother's and my brain both have tokens of a type of correlate for red experiences. Because science aims at generality, we aim at *types*-NCCs; but what we gather in research are *token*-NCCs. So there are sets of NCC-data (neural token/phenomenal token-tuples) and NCC-hypotheses (neural type/phenomenal type-tuples).

We have to state explicitly to *which type of experience* we are searching a correlate. My experience of the color oxblood *now* will only have *one* neural correlate, namely that minimal neural activation that was actually sufficient for me seeing oxblood. But we may talk of broader and broader types, e.g., red experiences, color experiences, visual experiences, sensory experiences, experience while being awake, and so on. The most general correlate we may be interested in is what distinguishes all conscious mental activity from un- or preconscious mental activity. Call this the *general* NCC (or neural correlate of *general* consciousness).

If the goal of a neuroscience of consciousness is to find Chalmers-NCCs, then we intend to find minimal neural systems which can be activated in such a way that this activation is sufficient (but not necessary) for some experience. I argue that this is not what we ought to search for because it has several systemic disadvantages (section 2). If there is a way to operationalize “NCC” without running into these disadvantages while upholding mere-sufficiency and minimality, we ought to prefer this operationalization (section 3).

## 2. Four reasons why chalmers's operationalization is unsatisfactory

Say the main goal of a neuroscience of consciousness really was finding Chalmers-NCCs. What would and wouldn't we gain from this project? If the acceptability of operationalizations hinges in part on their pragmatic value, and if we gain too little from knowing just Chalmers-NCCs, we may want to search for something other than Chalmers-NCCs.

In the following, I take Chalmers's operationalization *literal* for the sake of the argument. Let us imagine a state where we just knew which neural entities fulfil the conditions for being Chalmers-NCCs. What wouldn't we get?

### 2.1. Problem 1: neural correlates of capabilities or of occurrences?

According to Chalmers's operationalization, a neural system is a Chalmers-NCC *in virtue of* some of its possible states. But it is the neural *system* which is the Chalmers-NCC, not any of its states. However, systems persist independently of the individual states they are in. (My washing machine, for example, exists independently of whether it is in the off-, colored-, or lingerie-setting.) Therefore, an organism *o* may possess a Chalmers-NCC for an experience *e* (e.g., pain) even if it is not in the state which is sufficient for *e*. Knowing that *o*'s nervous system has a Chalmers-NCC for *e* does not help then to determine whether *o* currently has this kind of *e*-experience (i.e., feels pain or not). In order to determine whether *o* feels pain, we would need to know whether a Chalmers-NCC is in the specific pain-conducing state, the state that is sufficient for pain experiences.

Systems also may possess states which they never reach. (My washing machine, for example, has a setting for lingerie; but I never used it, so it never was in that state.) Consider then an extreme case: some organism *o*'s nervous system has a minimal subsystem *N*_*e*_. *N*_*e*_ is a Chalmers-NCC for the experience of tasting jackfruit in virtue of a state *n*_*e*_ of *N*_*e*_ which is sufficient for such jackfruit-experiences. By mere coincidence, *N*_*e*_ never was in state *n*_*e*_ during the whole of *o*'s existence—*o* never had any experience of tasting jackfruit.^8^ However, taking Chalmers's operationalization literally, *o* still possesses a Chalmers-NCC of *experiencing the taste of jackfruit*—even though *it never had or will have an experience of that sort*. So in accord with Chalmers's operationalization, organisms can have neural correlates for never occurring experiences. The Chalmers-NCC is therefore *not* a correlate of the experiences themselves—it cannot be because systems and experiences are indifferent ontological categories with different beginnings, ends, and dynamics. At most, the Chalmers-NCC is a correlate of the *capability to have* such experiences.

If the Chalmers-NCC is only an operationalization of the capability to have certain experiences, it may have some disadvantages in application. Because in some of the most pressing cases of application, what we really are interested in are the correlates of *occurring* experiences: in monitoring anaesthesia, we want to know whether this patient *now* feels pains, not whether she *can* experience pains; in comatose care, we want to know whether this patient suffers *now*, not whether she is capable of suffering at all; in animal welfare, we want to know whether dogs feel pain *during* castration, not whether they *can* feel pain in general. Knowing that some organism has a neural pain matrix which *can* bring about pain experiences does not solve these issues. We want to know *when* this neural subsystem is in the state in question in order to use neural activation as evidence for that organism being in pain.

This shortcoming may be easily fixed by making the neural states or processes themselves the NCC, not the system which is in these states.

### 2.2. Problem 2: neural evidence for ruling out experiences?

Say we consider states rather than systems as NCCs. Then, detecting a certain neural activation *n* may be treated as evidence for some organism currently having the experience for which *n* is an NCC. For example, if we detect state *n*_*e*_ in a patient under anaesthesia—*n*_*e*_ being a neural correlate of pain—, we gather evidence that this patient is currently experiencing pain.

But in some cases of application, we may want to be more cautious: in anaesthesia, we may want to *rule out* that a patient is currently experiencing pain; in comatose care, we may want be certain that a patient is *not* experiencing pain; in animal ethics, we may want to ensure that castration does *not* hurt the dog.

Ruling out the occurrence of experiences based on neural evidence is blocked by the mere-sufficiency-constraint: if I know that *A* is sufficient for *B*, I cannot rule out that *B* is absent if *A* is absent. If I were to assume the absence of *B* due to the absence of *A*, I would commit the fallacy of negating the antecedent. Say spitting on the street is sufficient for a fine; if you know that I did not spit on the street, you still cannot infer that I did not get fined—I might be fined due to jaywalking or insulting a policewoman. Several circumstances can be sufficient for something to happen. If we presume that a patient or organism *is not* feeling (or *cannot* feel) pain, because a specific minimally sufficient neural state $n_{e}^{1}$ for pain cannot be detected, we would commit the same fallacy.^9^ Because any neural state can only count as *merely sufficient* for an experience like pain, we can never rule out that some organism feels pain—because there could always be some other state $n_{e}^{2}$ that the organism is in, which is also sufficient for pain. Due to the mere-sufficiency-constraint, we can never rule out that somebody currently is undergoing some experience based on neural evidence.

One might think that this is too quick: we might rule out that an organism has a certain experience like pain if we fail to detect *all* Chalmers-NCCs for pain. But we will never be in the position where we know that we have detected all neural states minimally sufficient for pain. In order to do so, we would need to know what is neurally *necessary* for pain. But this seems to go against the mere-sufficiency-constraint. Because the mere-sufficiency constraint is reasonable to uphold, we can never rule out that some organism currently has any arbitrary experience based solely on neural evidence. This would severely limit the applicability of NCC-research.

### 2.3. Problem 3: multiplicity or uniqueness of NCCs?

An operationalization can be artificial—it does not need to capture any real usage, but instead establishes how to use a term. But if accepted, it ought to mirror what people intend to search for or how they talk about what is operationalized. Otherwise, there will be a mismatch between usage and operationalization. Some degree of deviation between operationalization and usage is acceptable. However, concerning the Chalmers-NCC, there might be too much deviation between operationalization and actual use.

Chalmers's operationalization allows many Chalmers-NCCs for the same conscious experience (each being sufficient for that experience). So any Chalmers-NCC can only be addressed as *a* NCC, not *the* NCC. Talking of *the* NCC, however, is a common way of speaking (see e.g., Crick and Koch (1998, p. 35), Crick and Koch (2003), Block (2005, p. 49), Lamme (2006), Singer (2016)).

It is also common to criticise a proposal as *not* being *the NCC proper*: Ned Block argues that there is only *one* NCC for visual phenomenality (Block, 1998, 2005); Crick and Koch (1995) hold that only those activations which are directly projected to the prefrontal cortex are *the* NCCs of visual consciousness, with other activations being in its “penumbra”.^10^ Even though the activation of some neurons will correlate strongly with experience, this activation does not count as part of *the* NCC.

This talk of *the* NCC suggests uniqueness, but Chalmers's operationalization does not capture this uniqueness due to the mere-sufficiency-constraint.^11^ If we want to capture the intended uniqueness of NCCs, we have to amend Chalmers's operationalization. But uniqueness can only be established by providing necessary conditions, which seems to be ruled out by the mere-sufficiency-constraint.

### 2.4. Problem 4: experimentum individualis or experimentum crucis?

For any scientific field, being able to comparatively test hypotheses is a methodological desideratum: given some data, which of an array of hypotheses is *best* supported by this data? An *experimentum crucis* is an ideal form of comparative testing. In an *experimentum crucis*, we do not only seek to find which hypothesis is *best* supported, but also shrink the array of competing hypotheses by *rejecting* some: given some data, which hypothesis is best supported *and which are falsified*? Chalmers's operationalization seems to block us from using this valuable methodological tool for NCC-research.

When do hypotheses compete? First, they have to be general enough in order to cover the same ground, i.e., concern type-correlates. There are some theories of consciousness which implicate type-correlates. Any such theory can only count as a *general* theory of consciousness if it intends to implicate all specific NCCs or the general NCC. ^12^ If there are two hypotheses *H*_1_ and *H*_2_ where both claim to be such general theories of consciousness, they only count as different if they are not co-extensional. They are not co-extensional if and only if they implicate diverging sets of neural activation being NCCs. So for each of these theories, there is at least one potential NCC uniquely implicated by it. But then, not *both* can be general and true hypotheses: Either each fails to implicate a proper NCC (by which they are not general), or one implicates a false NCC (wherefore it is not true). So either we believe that neither theory is general—both are only part of the full story; or we believe that at least one is false. We may try to figure out which one is most likely the true and general theory of consciousness by finding evidence that forces us to reject one but not the other. One way to do so effectively is by an *experimentum crucis*.

We may reject one of the competing theories either by evaluating the plausibility of each in itself; or we can evaluate them in comparison. If we evaluate each theory on its own, we would see for each theory if the NCCs it implicates actually do correlate with the experiences in question. If these implicated NCCs do correlate with experiences, then that theory becomes corroborated; if they fail to correlate with experiences, that theory looses credibility. But this is a laborious way of testing, because for each candidate, we have to go through all implicated NCCs.

A more efficient way may be the *experimentum crucis*. Empirical hypotheses, theories, paradigms and so on (abbreviated as *H*) describe ways the world could be: if *H* is true, such-and-such is the case. But *H* also *restricts* the ways the world could be: if *H* is true, such-and-such cannot be the case. Experiments can be understood as manipulating the world to see whether it conforms with some specific *H*, i.e., whether *H* describes not only a possibility but actuality. Let us individuate hypotheses by those “ways the world could be” that they are compatible with.^13^ Any hypothesis can then be associated with a set of possible scenarios.^14^ Any possibility not in this set counts as *incompatible* with this hypothesis. For example, Aristotle's hypothesis about the acceleration of falling objects can be associated with all those possible scenarios where a heavy object falls faster than a lighter one; Galilei's hypothesis is associated with those where all objects fall equally fast; each associated set of “ways the world could be” is incompatible with the other.

**Figure 1:**
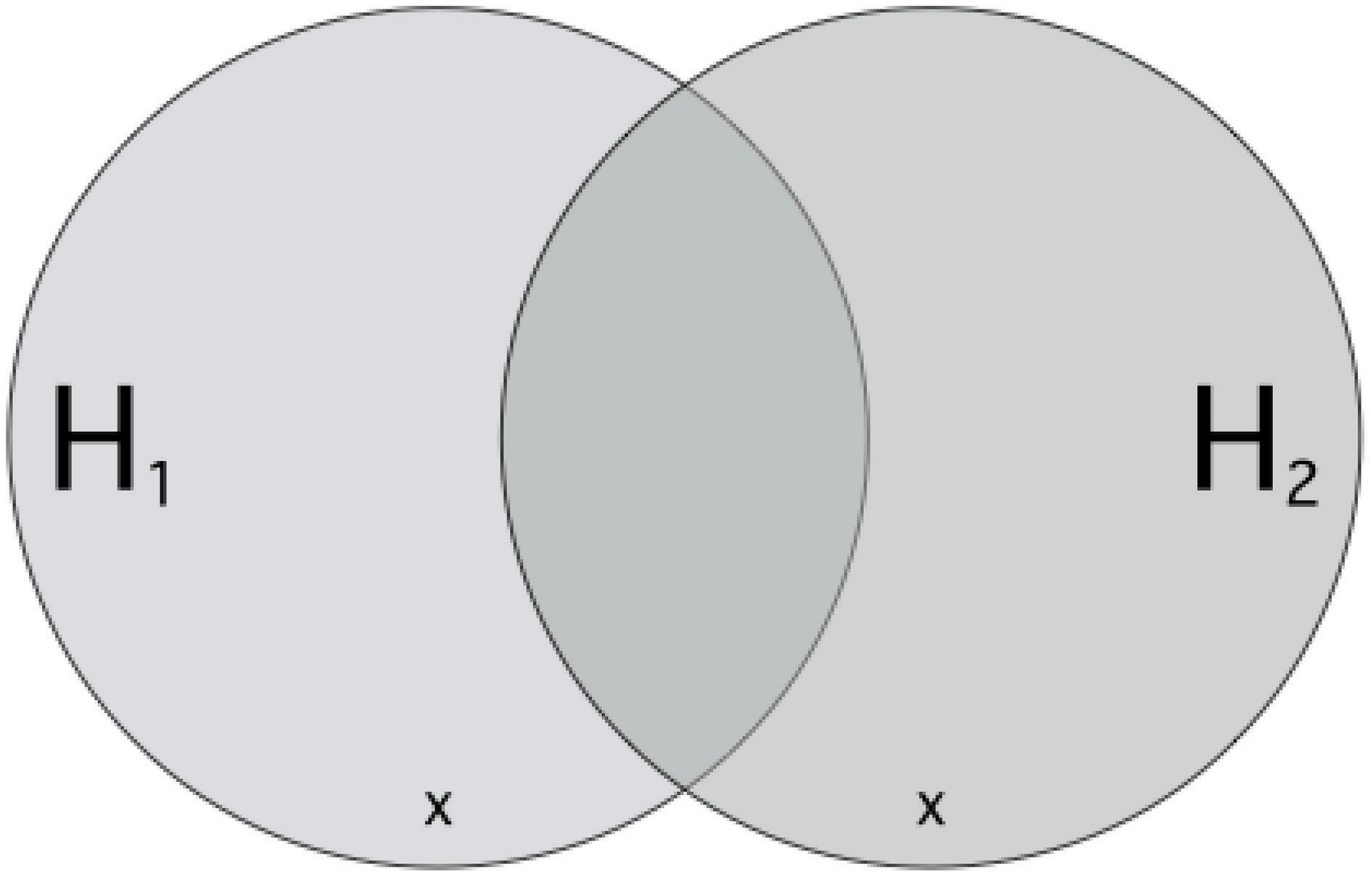
**An Illustration of Inter-Hypothetic Competition**. Let each point inside the border stand for a way the world could be, a possible scenario. Each circle picks out those scenarios compatible (i.e., not excluded) with some hypothesis. Hypotheses *H*_1_ and *H*_2_ stand in inter-hypothetic competition because for each, there are scenarios compatible with one but incompatible with the other. × s mark exemplary decisive events (*e*_Δ_). If such an *e*_Δ_ is observed, the credibility of one hypothesis rises while the other simultaneously declines.

Understood in this way, hypotheses *H*_1_ and *H*_2_ stand in competition if and only if they each share at most some but certainly not all ways the world could be with each other^15^. Then, there are some scenarios that are compatible with one but incompatible with the other and vice versa (see Figure 1). If something like this is the case, then one cannot rationally believe both hypotheses because they are mutually exclusive: for example, if *H*_1_ where Aristotle's theory about the acceleration of falling objects, and *H*_2_ were Galilei's, then one cannot believe both together: heavy objects cannot fall as fast as light one's and *simultaneously* faster.

If two hypotheses compete in this way, they enable *experimenta crucis* which clarify our doxastic allegiances, i.e., determine which of these hypotheses we *ought* to believe. The goal of an *experimentum crucis* is to investigate a scenario predicted by one theory but excluded by the other as being a way the world could be. If the world behaves as predicted, then this *simultaneously* corroborates the predicting hypothesis and discredits its competitor. The events investigated in these *experimenta crucis* are therefore *decisive* (*e*_Δ_, examples of which are marked by × in Figure 1), because they boost the credibility of one hypothesis while deflating the credibility of the other. In the ideal case, they decide which of the two hypotheses must be preferred.^16^

Some hypotheses in the neuroscience of consciousness look as if they allow for *experimenta crucis*. However, if these theories only implicate Chalmers-NCCs, an experimentum crucis is blocked: any Chalmers-NCC is merely sufficient for an experience, and therefore does not exclude any other neural state from also being a Chalmers-NCC. Given that we want to have a way of comparatively testing different general theories of consciousness, it would be beneficial to alter our operationalization of “NCC,” ideally in such a way that it allows for *experimenta crucis*.

#### 2.4.1. A possible example of inter-hypothetic competition in NCC-research?

Are there any examples of competing hypotheses in the history of NCC-research? There are two conditions for competition: first, the two hypotheses must make or imply claims about neural correlates for the same type of experience. (NCC-hypotheses for color vision do not directly compete with NCC-hypotheses for audition.) So our best bet to find competing hypotheses is among those aiming at *general* consciousness, i.e., hypotheses that focus on what distinguishes arbitrary conscious from unconscious mental processes. Second, the two hypotheses must differ in at least one ascription of consciousness to some specific neural event in a system. If the same ascriptions are implied by two hypotheses, they are empirically and extensionally undistinguishable.

Two hypotheses, which likely fulfil these two conditions, are the Recurrent Processing Hypothesis (RPH), according to which the occurrence of consciousness depends on recurrent processing (see Supèr et al., 2001; Lamme, 2004, 2006); and the Integrated Information Hypothesis (IIH), according to which the occurrence of consciousness depends on integrated information (see e.g., Tononi, 2004, 2008; Balduzzi and Tononi, 2009; Aleksander and Gamez, 2011; Oizumi et al., 2014). (As I treat both hypotheses as examples for a more general point—methodological problems raised by Chalmers's operationalization—it is excusable that I focus mainly on IIH's early iterations up to 2009.)^17^ Both Lamme's and Tononi's theories can be read as *general* neuroscientific theories of consciousness because both intend to indicate which neural activations come with (arbitrary) conscious experiences and may be used as neural evidence for consciousness: if some neural activation in a neural system *N* has feature *F* (here: processes using recurrent connections or integrating information), then this suffices for that system being conscious. All and only those neural activations with *F* are NCCs, but Lamme and Tononi differ on the NCC-making feature in question.^18^

According to IIH, if some system *s* generates some degree of integrated information (Φ > 0), then *s* is phenomenally conscious—and if Φ = 0, *s* is unconscious. Tononi argues by analogy: phenomenologically, our experiences are governed by differentiation and integration. We can have a wide variety of experiences (the sound of a loved one's whisper, the pangs of pain, the pulsing pleasures of orgasms, the rich vibrancy of purple, and so on); but whatever experiences we are having in a moment, all are incorporated in a holistic *me-being-in-the-world-with-this-and-that-happening-now* (see e.g., Brentano, 1995; Heidegger, 1986; Bayne, 2010). Tononi holds that an analogous feature can be defined in informational terms: the changes of some part of a system not only generate information by themselves, but also about other parts of the system. This information generation may be so tightly entangled in certain complexes that such a complex *as a whole* generates *additional* information. Some ways to calculate such “integrated information” (Φ) have been presented, for example, in Tononi (2004, 2008) and Balduzzi and Tononi (2009)^19^. (How to calculate Φ has changed in version 3.0 of the theory (Oizumi et al., 2014) such that a competition with RPH is less obvious. IIH-2014 will largely be ignored here).

According to RPH, if some system *s* engages in recurrent processing (i.e., feeds information back from latter stages of processing to earlier stages), then *s* is phenomenally conscious—and if there is no recurrent processing going on in *s*, then *s* is unconscious. While Tononi starts from phenomenology, Lamme (2004, p. 867f) bases this hypothesis on several observations in vision research. First, if we present two stimuli very shortly after one another (e.g., 40 ms delay), the first stimulus still causes a feed-forward sweep of activation, but is not consciously seen (Enns and Di Lollo, 2000). Recurrent processing of the first stimulus is suppressed by the feed-forward sweep elicited by the second stimulus, producing a kind of *backward masking* (Lamme and Roelfsma, 2000; Lamme et al., 2002). Second, in a *binocular switching paradigm*, we present two stimuli, one to each eye simultaneously. The features that distinguishes objects from their backgrounds are switched from one stimulus to the other. For example, one eye sees a red face on a green background and the other a green face on a red background. Each stimulus is processed feed-forward; but in each moment, only one is consciously seen—namely the one being recurrently processed. The other, purely feed-forward-processed stimulus remains invisible (Moutoussis and Zeki, 2002). Third, if we disrupt neural activation with transcranial magnetic stimulation (TMS) *after* a stimulus has evoked a feed-forward sweep, the stimulus is rendered invisible (Corthout et al., 2000). So if there is no recurrent processing, the stimulus lacks phenomenal character. Fourth, feed-forward processing can still be recorded in anaesthetized animals, who are presumed unconscious. Recurrent processing, however, is drastically reduced during anaesthesia (Lamme et al., 1998). Fifth, in figure-ground-segregation tasks, if stimuli are reported as “not seen” although they are present, feed-forward activation is still happening: the stimuli cause neural activation and are processed. According to Lamme(2004, p. 868), a “neural correlate of figure-ground segregation, probably mediated by recurrent interactions between V1 and extra-striate areas, and present when stimuli are seen, is, however, fully suppressed when stimuli are not seen (Supèr et al., 2001).” So there seems to be some correlation between visual experiences and recurrent activation. Lamme extends these insights to phenomenality in general: recurrent activation is the general neural process by which a system brings about consciousness.^20^

RPH and IIH cover the same subject matter: which systems in the world are conscious under which circumstances—and which aren't. If we want to construe an *experimentum crucis* between these two theories, we might want to search for systems to which conscious experiences are ascribed by one hypothesis but not by the other. We may then investigate the systems in question for the presence of consciousness. If we can detect consciousness reliably, we may then find confirmation of one hypothesis and disconfirmation for the other in such “decisive systems.” Such systems can be found.

**Figure 2:**
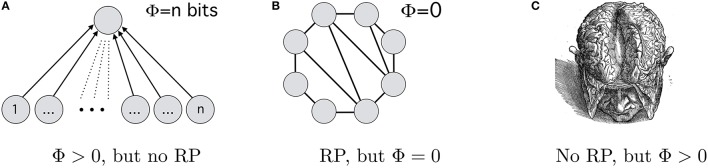
**Three decisive instances**. According to RPH, **(A)** and an inactive (but activatable) brain **(C)** are unconscious, while **(B)** may bring about consciousness; according to IIH-2008, **(B)** is unconscious and **(A)** is conscious, while **(C)** can be conscious in all of IIH's iterations.

According to the 2008-version of IIH (Tononi, 2008, p. 235), if we increase the number of neurons that all need to be active in order to excite an *and*-gate neuron in a higher level, then each lower level neuron we add increases the Φ-value of this system (see Figure 2A). But such a system would not have any recurrent connections. IIH in its 2008-version therefore ascribes experiences to the system, while RPH would deny it consciousness.^21^ Second, Tononi (2008, p. 225) presents a system which generates no integrated information (Φ = 0) if all its neurons are active. We can count some of the connections as recurrent. (A similar system can be found in (Oizumi et al., 2014, 14) where Φ borders on 0; similarly, subsystems of a system can have Φ = 0 in version 3.0 despite possible recurrent processing.) Thus, if all neurons in this system are active, then there is recurrent processing going on but Φ = 0 (see Figure 2B). Thus, IIH-2008 ascribes no consciousness to a system, where RPH attests it to be conscious. Third, consider that a 0 carries just as much information as a 1—the information carried by 0100001 in binary differs from the information carried by 11. This applies equally to a brain: inactivation of a neuron carries information, e.g. about the absence of a stimulus. If so, then an *inactive* brain may have Φ > 0 and therefore be conscious according to all versions of the IIH.^22^ Obviously, there would be no recurrent processing going on. Then, IIH in any iteration would ascribe consciousness to an inactive (but revivable) brain while RPH would not.

Both IIH and RPH can be considered general theories of consciousness. Both have the same subject matter. Both implicate certain types of activation as being NCCs, neutrally construed: each makes mutually excluding predictions about when systems are conscious (and when not). It seems therefore that there is potential for inter-hypothetic conflict: either both are only partial theories of consciousness, or (if they are not partial theories of consciousness) they stand in competition. Whether consciousness arises in these systems determines the outcome of an *experimentum crucis* between IIH and RPH: for each theory, there is at least one way the world could be (systems like 2a–2c being conscious or not) that lowers the credibility of one hypothesis and simultaneously raises the credibility of the other. It seems that *experimenta crucis* are possible in a neuroscience of consciousness.

#### 2.4.2. The problem: chalmers's operationalization

RPH and IIH seem to differ on which neural events can be considered NCCs, and it seems that if we take them to be general theories of consciousness (which they are intended to be), we ought to consider them as standing in competition. Can we perform an *experimentum crucis* if we consider each theory as implicating only Chalmers-NCCs?

No, because recurrent processing *as well as* Φ > 0 can *simultaneously* be Chalmers-NCCs: if an inactive brain is consciousness, then Φ > 0 is sufficient for consciousness; but this does not speak *against* recurrent processing being sufficient for consciousness as well. Having Gouda is sufficient for serving cheese for desert, but this does not rule out that Stilton is sufficient as well. Despite appearance, if these theories only implicate Chalmers-NCCs, none of the crucial instances in Figure 2 suffices for an *experimentum crucis*. Although both IIH and RPH make diverging predictions, there is no inter-hypothetic competition between them—and there cannot be any if they only implicate Chalmers-NCCs.^23^

There are several ways how one may react to this lack of inter-hypothetic competition. First, one might see both IIH and RPH as special cases of a different, more overarching theory. This means that we give up counting each as a *general* theory of consciousness. This motivates an additional search for the overarching theory. Second, one might think that consciousness is just not governed by natural laws. So each of the two features (recurrent processing and Φ > 0) correlates contingently with consciousness in some cases, but not in others, and we cannot say why. This mysterian option must be unattractive to any naturalist, and it would have terrible practical consequences as well: we could never be certain that anaesthesia works on you, because consciousness does not follow any rules predictably. It is the worst option for anybody interested in a *natural science* of consciousness. Third, we might reconsider whether these theories implicate only Chalmers-NCCs, or what the relation between Chalmers-NCCs and general theories of consciousness ought to be. Because Chalmers's operationalization has further drawbacks, as I argued above, I explore this third option.

## 3. An alternative account: toward an NCC2.0

I presented four shortcomings of Chalmers's operationalization of “NCC”. Because any operationalization is based on a decision, these drawbacks might lead us to rethink whether we made the right decision in preferring Chalmers's operationalization over possible alternatives. Even if Chalmers captured some plausible meaning of “NCC”, it seems that “NCC” ought to refer to something else, something which does not give rise to these four issues, something like an NCC2.0.

Here is the challenge. On the one hand, any NCC2.0 ought to *uphold the mere-sufficiency-constraint* in order to (i) be able to account for neural redundancy and neural plasticity, (ii) be compatible with the possibility of artificial experiencers, silicon brain prostheses, or extended neural+*x*-bases of consciousness. On the other hand, in order to overcome some of the problems of the Chalmers-NCC, it seems that we have to introduce necessity somewhere. Only then can we allow for inter-hypothetic competition, and only then can we apply our best NCC-hypotheses to cases where we want to *rule out* that an organism currently is feeling something based on neural evidence. The crux for any NCC-operationalization is then to balance mere sufficiency of neural activation for conscious experience with some kind of necessity of neural activation for consciousness.

We might take a hint on how to achieve this balance from Chalmers himself. In *The Conscious Mind* (Chalmers, 1996, p. 275), he advocates a form of “nonreductive functionalism, on which functional organization suffices for conscious experience with natural necessity. […] We have narrowed down the relevant properties in the supervenience base to organizational properties: […] for every physical system that gives rise to conscious experience, there is some functional organization *F* realized by the system, such that it is naturally necessary that any system that realizes *F* will have identical conscious experiences.” Despite any neural event being only sufficient, there is some NCC-marking feature *F*: anything that is an NCC is *F*, anything that is not an NCC lacks *F*. While *F* does not make some neural activation an NCC with conceptual, metaphysical, or epistemic necessity, we may presume that something like a unique NCC-marking feature exists with natural necessity. We may find this NCC-signature *F a posteriori*. And, most plausibly, this feature is functional.

Building on these thoughts, my proposal is the following: we accept mere sufficiency of the neural for the phenomenal on the level of *tokens*, but claim necessity of the neural for the phenomenal on the level of *types*. An operationalization then might read:

**NCC2.0:** An NCC2.0 of a phenomenal *type P* is a *type* of neural event or process *N* such that there is a mapping, where (i) each neural token *n*_*i*_ of *N* is minimally sufficient for a phenomenal token *p*_*i*_ of *P*,^24^ and (ii) where all and only neural tokens of *N* instantiate a feature-bundle 𝔽, such that 𝔽 is a (naturally) *necessary* condition for being an NCC of *P*.^25^

The NCC2.0 explicitly distinguishes between token- and type-correlates. Although token-correlates are *events*, not systems, they share a lot with the classical Chalmers-NCCs: neural events ought to be minimal but sufficient, but no token is strictly necessary for an experience. Thus, we preserve the minimality- and mere-sufficiency-constraint on the token level. On the level of types, we reject the mere-sufficiency-constraint—and the minimality-constraint follows trivially from the newly introduced natural-necessity-constraint.

The NCC2.0 is a *metaphysical* operationalization. It tells us which entities out there in the world ought to count as NCC2.0s. By accepting it, we presuppose that all individual neural events bringing about experiences share a commonality which distinguishes them from all other events that don't bring about experiences. (*Methodologically*, NCC2.0-hypotheses can be differentiated by the neural feature-bundles they mark out as being the NCC-making feature, e.g. recurrent processing, or Φ > 0). So this operationalization comes with the baggage of an ontological commitment: in order for NCC2.0s to exist, there must be some form of bijective or one-to-one-type-type-mapping. Even though it is likely that something like that is the case, we cannot be certain that such a mapping exists. We must take the following two options seriously. First, consciousness may cross-cut any reasonable way to type the natural world such that there is no ontological one-to-one-type-type-mapping between neural and phenomenal types. This would be the case if, for example, strong 4*E*-accounts (according to which consciousness is extended, embedded, enactive, or embodied) are right. Then, consciousness has no fixed neural basis, and therefore there is no one neural type corresponding to any given phenomenal type. Second, there might be a bijective type-type-mapping, but the neural types could be so weirdly constructed and complicated that we would likely never find the right way of typing neural events, making the type-type-mapping epistemically impenetrable. Neither option can and should be excluded *a priori*.

But even if these options were the case, the NCC2.0 is a reasonable way to phrase the goals of a non-4*E*-neuroscience of consciousness. If, after a long stretch of empirical neuroscientific research, we do not find anything in the brain fitting this operationalization, then we have reason to believe that consciousness is either not a natural or simple phenomenon, or that it has no exclusively neural correlate. There may then be a justified shift from a pure neuroscience of consciousness to 4*E*-accounts in the long run.

Even if strong 4*E*-accounts are wrong and most phenomenal experiences have a corresponding neural type, the NCC2.0 is open for *partial* contributions from 4*E*-accounts. It might be that for *some* phenomenal experiences, neural activation suffices, but for *some other* phenomenal experiences, external factors have to contribute. For example, the phenomenality of a stable visual experience might be something that relies strongly on external factors, because in dreams, where visual experiences are elicited solely by the brain, visual experiences are much more fleeting and haphazard. The NCC2.0 allows for such contributions, because all it claims is that all *neural* types whose tokens suffice for phenomenal tokens will share a common feature(-bundle) 𝔽. This is compatible with the idea that there might be *non*-neural- or neural+*x*-tokens which are sufficient for experiences. Incorporating such data might hint at more abstract properties being the intersubjectively available marker-bundle of conscious experience, e.g.,—as Chalmers predicts—functional or computational properties.

So there are some initial perks of this operationalization. But does it avoid the four problems raised against Chalmers's operationalization? Concerning the first problem: because some neural event tokens are what correlates with phenomenal event tokens, these neural events are token-correlates of *occurring* experiences; thus, any organism that never had an experience *x* will fail to have any *x*-corresponding neural token-correlate. Concerning the second problem: because the feature-bundle 𝔽 would count as a necessary condition for some neural event being a neural correlate, failing to detect any 𝔽s marked out by our best corroborated theories in some organism *o*—despite our best efforts—would license the belief that *o* is not having the experience that 𝔽 marks. Knowing that *o* is incapable of instantiating 𝔽, we are licensed to rule out that *o* is at all capable of having the experience in question. Concerning the third problem: because 𝔽 is what makes all neural tokens having 𝔽 NCCs, we can say that we search for *the* NCC2.0 on the type level—the feature-bundle 𝔽 that all and only token-NCCs share. We can presume that there is one natural, unique way of typing NCCs suggested by how we refer to 𝔽. (Still, there are many NCCs on the token level.) Concerning the fourth problem, we would need to answer first: how would the NCC2.0 shape research, data gathering and its relation to theory building?

Let us focus, first, on the collection of correlation-data. Most neuroscientific theories of consciousness, like Lamme's, start from bottom-up data, consisting of detections of individual occurrences of phenomenal experiences (*P*-tokens: *p*_1_ at time *t*_1_, *p*_2_ at *t*_2_, … ) and detections of simultaneously occurring individual neural events (*N*-tokens: *n*_1_ at *t*_1_, *n*_2_ at *t*_2_, … ). Each neural token here is a neural token-correlate of a token *P*-experience. Our collected data-points will share numerous features—and we cannot ensure that any collected data-point will be minimal. In accord with the NCC2.0, we presume *pre-theoretically* that there is a unique and specifiable corresponding type of neural activation associated with that type of *P*-experiences. This neural type is the *P*-type's neural *type*-correlate. Our goal, however, is to find out which feature-bundle marks out all and only minimal neural token-correlates. So we hypothesize and test in order to find *a posteriori* the *right* feature-bundle.

Generally, every type can be associated with a type-making property or set of properties or a property bundle. Our goal is to find the bundle of *neural* features that all and only neural token-correlates of occurring phenomenal events share. How might we do this? Consider, as an illustration, numbers being drawn from a pot. We draw 1, 2, 3, 4, and 3. If we were to use the type-making feature *being an odd number* or *being an even number*, we would not cover all numbers in this set. We might therefore settle for something like *being a natural number* or *being smaller than 5*. We then test these two hypotheses by drawing additional numbers from the pot. If one of them were $\sqrt{2}$, we know which of our hypotheses is false—the pot does not contain only natural numbers. So we proceed in two steps: first, we construe competing hypotheses fitting our current set of data (e.g., *being smaller than 5* vs. *being larger than 0*); second, we predict further data, and test our hypotheses by the data they predict or prohibit. Rinse and repeat.

The same holds for NCC2.0-hypotheses: given the neural-token–phenomenal-token-tuples from our data (〈*p*_1_, *n*_1_〉;〈*p*_2_, *n*_2_〉;…), we look for what these neural tokens have in common and what distinguishes them from other neural tokens. Whatever bundle of neural features we choose, it must also apply to some possible neural events outside of our original data set. All NCC2.0-hypotheses then predict that every neural event that has the hypothesis-respective neural feature-bundle 𝔽 correlates with the chosen type of experience. This is the structure of NCC2.0-hypotheses.

**Figure 3:**
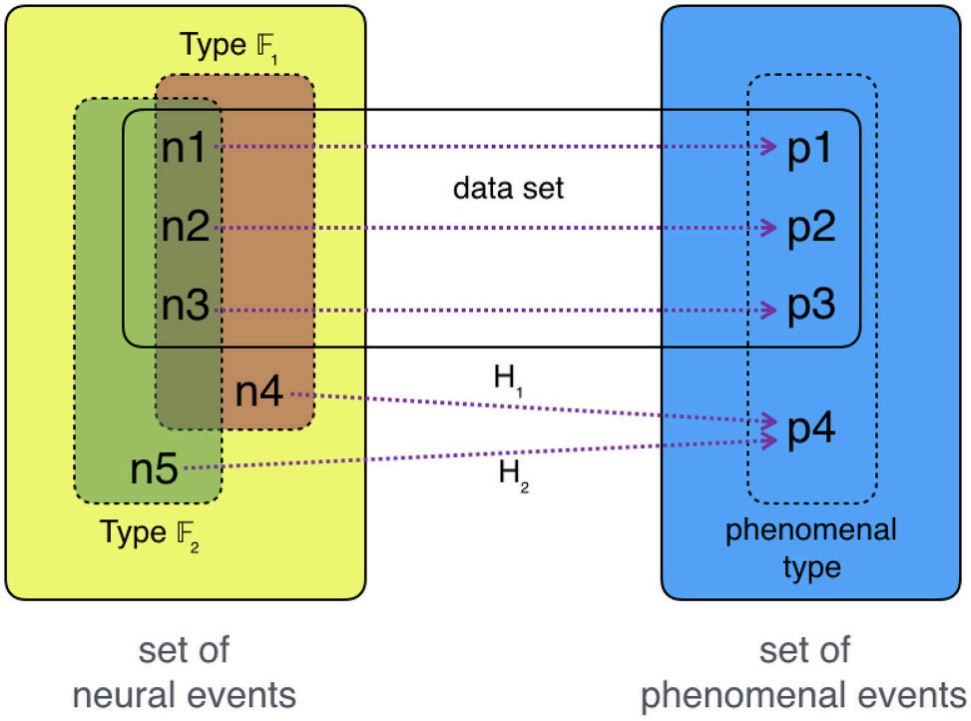
**Testing NCC2.0-hypotheses**. Our set of recorded data consists of *n*_1_, *n*_2_, *n*_3_ correlating with experiences *p*_1_, *p*_2_, *p*_3_ in the following way: 〈*n*_1_, *p*_1_〉;〈*n*_2_, *p*_2_〉;〈*n*_3_, *p*_3_〉. The phenomenal occurrences *p*_1_, *p*_2_, *p*_3_ are tokens of a phenomenal type *P*. The neural occurrences *n*_1_, *n*_2_, *n*_3_ can be “typed” in different ways: they share feature-bundle 𝔽_1_ as well as 𝔽_2_. This allows us to formulate two hypotheses, *H*_1_ and *H*_2_. We test these hypotheses by looking at neural events that instantiate one feature-bundle but not the other, here *n*_4_ and *n*_5_. Checking whether consciousness occurs in any of these two instances is an *experimentum crucis* between *H*_1_ and *H*_2_: if the organism is in *n*_4_ while it experiences a token of *P*, namely *p*_4_, then 𝔽_1_ is the likely NCC-marker for *P*-experiences—thus, *H*_1_ presents the most credible candidate for an NCC2.0 in comparison with *H*_2_.

There are two types of testing such NCC2.0-hypotheses. First, we might look for neural states $n_{y}^{1},n_{y}^{2},\ldots$ that *do not* instantiate 𝔽_*x*_. If $n_{y}^{1},n_{y}^{2},\ldots$ actually do not correlate with phenomenal tokens of type *P*, then we see this as corroboration that 𝔽_*x*_ marks all neural states sufficient for *P*-experiences. If $n_{y}^{1},n_{y}^{2},\ldots$ *do* correlate with some *P*-token-experiences, then this undermines the hypothesis that 𝔽_*x*_ marks all neural states sufficient for *P*-experiences. Any new NCC2.0-hypothesis must then take account of $n_{y}^{1},n_{y}^{2},\ldots$ correlating with *P*-experiences—the NCC-making feature must then be one shared by all neural 𝔽_*x*_-states as well as $n_{y}^{1},n_{y}^{2},\ldots$. Second, we might look for neural states $n_{x}^{1},n_{x}^{2},\ldots$ that *do* instantiate 𝔽_*x*_. If $n_{x}^{1},n_{x}^{2},\ldots$ do correlate with some *P*-token-experiences, then we see this as corroboration that 𝔽_*x*_ is an (*a posteriori*) necessary feature of all neural events sufficient for *P*-experiences—*only* token-correlates of *P*-experiences instantiate 𝔽. If $n_{x}^{1},n_{x}^{2},\ldots$ *does not* correlate with some *P*-token-experiences, then this undermines the hypothesis that 𝔽 is a necessary feature of all neural events sufficient for *P*-experiences. (See Figure 3 for an illustration.)

If some hypothesis H^*^ (stating some 𝔽^*^ as a the NCC-marking feature-bundle) becomes highly corroborated by a long progression of successful predictions and refutations, we may use H^*^ to check whether some organism *o* (i) is currently conscious by checking whether *o*'s nervous system is in a state instantiating 𝔽^*^, (ii) is unconscious by checking whether *o*'s nervous system is not in a state instantiating 𝔽^*^, (iii) cannot be conscious by checking whether any of *o*'s neural subsystems is capable of being in a state instantiating 𝔽^*^. Our best NCC2.0-hypotheses would then allow us to justifiably claim, at least in principle, that it is more or less likely that some organism is conscious or not—something we cannot do if we focus solely on Chalmers-NCCs. This makes knowledge of NCC2.0 in principle applicable to cases like anaesthetic awareness or the question whether a specific comatose patient or a certain animals are conscious or not.

A question remains about which kinds or features we ought to prefer as NCC-making features. If we want to be open to the possibility that animals with different neuroanatomy (like cuttlefish) can be conscious, we should not focus too much on neuro*anatomical* features. We ought to prefer broadly neurobiological, -chemical, or even neurocomputational, -topological, or -organizational features. If we want to be open for contributions or applications to AI-research, we might not want to choose neurobiological or -chemical features, but instead focus solely on neurofunctional or -computational features—just as IIH and RPH do. If we want to be open to substantial contributions by 4*E*-accounts, we may allow for the possibility that some experiences need contributing external factors which a neural system is in itself incapable of producing.^26^ All this is compatible with a research guided by the NCC2.0. Chalmers himself seems to prefer functional and organizational features, underlining his openness for artificial experiencers or extended bases.

Coming back to *experimenta crucis*: if we consider RPH and IIH as implicating NCC2.0s and not Chalmers-NCCs, there is straightforward inter-hypothetic tension between them. Two hypotheses are differentiable by the feature-bundle they choose to mark token-NCCs—here, Φ > 0 or recurrent processing. We then look whether there can be neural events that instantiate one but not the other. Some likely candidates (at least for IIH up to 2009) have been presented in Figure 2. If there are no such cases of divergence for some hypotheses *H*_1_ and *H*_2_, then these two hypotheses are extensionally equivalent, and therefore can be treated alike. (For all practical purposes, *H*_1_ = *H*_2_). If there is an asymmetry—all instantiations of 𝔽_1_ are instantiations of 𝔽_2_ but not vice versa—, then we ought to check whether the narrower hypothesis holds. (For all practical purposes *H*_1_ implies *H*_2_). If there are symmetric cases of exclusion, as in the case of IIH and RPH, then we can perform classical *experimenta crucis*: we see which of the systems is and which is not conscious and thereby simultaneously raise the credibility of one hypothesis and lower the credibility of the other. The touchstone for NCC2.0-hypotheses is then the succession of their successful prediction.

Clearly, these ways of testing hinge on our ability to assess whether a given system is conscious or not *from the outside*. For example, we would need to determine whether an inactive brain is conscious or not in order to compare IIH and RPH in an *experimentum crucis*. Generally, this is *the* core methodological problem for theory testing in empirical consciousness studies. It deserves its own research, guided by the question: which kinds of evidences are adequate or reliable indicators for consciousness under which circumstances and to which degree? This challenge is not overcome by switching from the Chalmers-NCC to the NCC2.0. But with the NCC2.0, there is at least no *fundamental* or *conceptual* problem for testing and comparing general theories of consciousness, just a common methodological one. The neuroscience of consciousness searching for NCC2.0s can in principle progress like any other science: by competing in the game of predictive fit.

## Author contributions

The author confirms being the sole contributor of this work and approved it for publication.

### Conflict of interest statement

The author declares that the research was conducted in the absence of any commercial or financial relationships that could be construed as a potential conflict of interest.

## Footnotes

^1^In some sense, this holds even for extreme extended or embodied accounts which implicate that *no* neural activation is *ever* sufficient for experiences. These theories therefore predict that nothing fulfils the criteria for an NCC. However, we ought to specify *first* what “NCC” ought to mean; *then* it might turn out that our best supported theory of consciousness implicates that there is nothing fitting this operationalization.

^2^It may be reasonable to presume that correlations approaching the extremes are the most relevant, because they have the most justificatory and predictive power. But even if events correlate to a relevant degree, then this does not show any meaningful connection: the divorce rate in Maine (US) correlates to a degree of +0.992558 with the per capita consumption of margarine in the US; the number of honey producing bee colonies in the US correlates to a degree of −0.933389 with its number of juvenile arrests for possession of marijuana (see http://tylervigen.com). Even strong correlations can arise by accident without implying any kind of relevance. For this reason, our knowledge of NCCs will not decide which sufficiently specific solution of the mind-body-relation is the case, because each can be made compatible with the NCCs we find by some *ad hoc* additions.

^3^Its first mention is in the philosophical journal *Mind* in an article by Marshall (1901). Afterwards, it is mentioned in the *Psychological Review* (Weiss, 1917, p. 360f), two years after J. B. Watson ended his editorship. Then, it turns up again in Sir William Mitchell's Gifford Lecture *The Place of Minds in the World* (Mitchell, 1933, p. 123f). See also Griffith (1967, p. 25). It is unclear whether Crick adopted the term from anybody. But while previous authors simply use the term, he is the first to give an elucidation of what might be meant by it.

^4^For authors accepting the possibility of non-biotic consciousness, see e.g., Gamez (2008); Holland and Goodman (2003); Metzinger (2004, 2013); Reggia (2013) or anybody publishing in the *International Journal of Machine Consciousness*.

^5^Think of a system composed of neurons *n*_1_, *n*_2_, *n*_3_. It is conceivable that the activation of *n*_1_ and *n*_2_ brings about a red experience *as well as* the activation of *n*_2_ and *n*_3_ *and* the simultaneous activation of all three. In the first case, the activation of *n*_3_ is redundant—if one would lesion *n*_3_, the organism could still experience red. But one could also lesion *n*_1_ without prohibiting the organism from experiencing red, because in the second case, *n*_1_ is redundant. However, one cannot lesion *both n*_1_ and *n*_3_ without the organism losing its capability of experiencing red—the activation of *n*_2_ by itself is not sufficient for red experiences. In this case, there is no single set of individual neurons sufficient *and necessary* for red-experiences. And because there is no reason to rule out such a mapping *a priori*, we should not subscribe to the necessity-constraint.

^6^Mapping is here best understood mathematically: there is a function such that some states of the neural system *N* are mapped onto states of consciousness. Chalmers, however, leaves it open whether the mapping ought to be understood as bijective, injective, surjective, or projective. Depending on which type of mapping we presume, different metaphysical positions are ruled out. But given that Chalmers leaves the specific type of mapping open, we might presume that these constraints on metaphysics can only be settled after the data is in. Given the presumptions of naturalism and the mere-sufficiency-constraint, however, a surjective mapping of states of *N* to states of consciousness would be the most natural contender.

^7^See also: “The Neural Correlates of Consciousness (NCC) can be defined as the minimal neuronal mechanisms jointly sufficient for any one specific conscious percept.” (Mormann and Koch, 2007). Interestingly, the reference “(Crick and Koch, 1990)” cited by Mormann and Koch (2007) does *not* contain this characterization. The minimality-constraint as well as the mere-sufficiency-constraint were both first introduced by Chalmers in 2000. A similar rendition of the NCC, again without mentioning Chalmers, can be found in Koch (2004) and Koch (2012 p. 42): “In the early 1990s, Francis Crick and I focused on what we called the *neural* (or *neuronal*) *correlates of consciousness* (NCC). We defined them as the minimal neural mechanisms jointly sufficient for any one specific percept.” However, they never defined “NCC” like this before Chalmers's article in 2000. Although there might be some informal precursors, the written record does not support these statements by Koch and colleagues. In a more malicious interpretation, it might be an instance of “editing out” philosophical contributions to the neuroscience of consciousness. According to Kuhn (2012), such “boundary work” (cf. Gieryn, 1983) is a sign of a science's transformation from a pre-paradigmatic stage to a “normal science.” Be that as it may, the missing reference to Chalmers's article suggests a misappropriation of credit.

^8^Maybe you are such a system.

^9^Rose (2002) has argued in this fallacious way for fishes being incapable of feeling pain: he writes that they cannot experience pain because they lack a cortex, and a cortex brings about experiences in us. Therefore, fishes cannot experience pain. However, if any neural correlate is merely sufficient for an experience, then some other neural structure may suffice for pain experiences. The lack of a cortex therefore does not rule out that fishes feel pain.

^10^“The NCC at any one time will only directly involve a fraction of all pyramidal cells, but this firing will influence many neurons that are not part of the NCC. These we call the ‘penumbra’.” (Crick and Koch, 2003, p. 124)

^11^But maybe these scientists speaking of *the* NCC thereby merely attest that some neural states are not *minimal* neural correlates? This is certainly true for the penumbra. However, the usage of “the” suggests that the proposed *minimal* NCC is seen as the *unique* neural correlate of that experience. Seeing any minimal NCC as unique would not be admissible if the proposed neural activations were considered to be Chalmers-NCCs.

^12^“NCC” in this section is used in a pre-operationalized way, if not indicated otherwise.

^13^This can be understood as an extensional way to individuate hypotheses. Clearly, two hypotheses *H*_1_ and *H*_2_ could be compatible with the same ways the world could be, but look differently. Maybe they use different formulas, and different looking calculations, different names, etc. but still are compatible with the same ways the world could be. These, however, shall not be of interest here, because there is no way to decide between these hypotheses *by experiment*. Here, I will restrict myself to hypotheses that stand in inter-hypothetic competition which can be resolved via some experiment or data-gathering.

^14^That is, they resemble propositions under some understanding of the term (cf. Montague, 1960).

^15^If they share no members, they do not even share the factual descriptions of reality. Either at least one is purely fictional or they simply range over different sections of reality: one might be an economic theory about asset price inflation, the other a theory about DNA-methylization. In such cases, they hardly compete, because one can believe both. However, there might be some hypotheses that do not overlap explicitly, but implicitly. A theory about motor control in mammals and another about motivated action might not look like they overlap, but they do because they can have incompatible implications.

^16^That is: if an ideally rational subject *s* compares *H*_1_ and *H*_2_ and if a decisive event *e*_Δ_ is observed, then the *s*'s credences (*P*_*s*_) should alter in the following way: ([*P*_*s*_(*H*_1_|*e*_Δ_) > *P*_*s*_(*H*_1_)] ∧ [*P*_*s*_(*H*_2_|*e*_Δ_) < *P*_*s*_(*H*_2_)]) ∨ ([*P*_*s*_(*H*_2_|*e*_Δ_) > *P*_*s*_(*H*_2_)] ∧ [*P*_*s*_(*H*_1_|*e*_Δ_) < *P*_*s*_(*H*_1_)]) or, simpler put: [*P*_*s*_(*H*_1_|*e*_Δ_) > *P*_*s*_(*H*_1_)] ≡ [*P*_*s*_(*H*_2_|*e*_Δ_) < *P*_*s*_(*H*_2_)]. The credibilities of *H*_1_ and *H*_2_ are entangled.

^17^The Integrated Information Hypothesis has changed considerably over time: there are different versions by Tononi, where the motivation for differences and divergences from earlier iterations are often unclear. In addition, there are several competing proposals to Tononi's on how to assess the amount of integrated information (Φ), e.g., Seth et al. (2006); Barrett and Seth (2011); Seth (2011); and Aleksander and Gamez (2011). I will focus mainly on Tononi's approach up to 2009 (Tononi, 2004, 2008; Balduzzi and Tononi, 2009). Some of what I say will not hold for newer iterations, e.g., Oizumi et al. (2014). But I merely discuss both theories as *examples* to illustrate a shortcoming of Chalmers's operationalization. It therefore suffices that there was some moment in the past where two general theories of consciousness competed. Between 2004 and 2009, we can be confident that such a competition was the case.

^18^Unlike many other proposals, neither theory refers explicitly to any specific type of experience (e.g., seeing color, hearing, feeling one's body) or to any anatomical or biochemical features. Instead, both see broadly computational, topological, or architectural features as crucial. Thus, their proposals—if tenable—might even apply to artificial systems.

^19^Here, the governing formula to calculate Φ for some system in state *x*_1_ is Φ(*x*_1_) = *ei*[(*X*_0_(*MIP, x*_1_) → *X*_0_(*mech, x*_1_)], where *ei* is the effective information, *X*_0_(*mech, x*_1_) denotes the repertoire of potential states given some mechanism governing that system and MIP stands for the minimal information partitions. Alternatives for assessing Φ have been proposed (Seth et al., 2006; Barrett and Seth, 2011; Seth, 2011; Aleksander and Gamez, 2011; Tononi, 2012a,b). For lack of space, these will ignored here. See also Edlund et al. (2011) as well as Joshi et al. (2013).

^20^“According to such empirical and theoretical arguments, [recurrent processing] is the key neural ingredient of consciousness. We could even define consciousness as recurrent processing.” (Lamme, 2006, p. 499). Taking the proposal that consciousness can be defined as recurrent processing at face value, recurrent processing would count as sufficient for consciousness.

^21^This does not apply anymore to the newest version of IIH: Oizumi et al. (2014, p. 21) present a highly complex feed-forward system, but where Φ^*MAX*^ = 0.

^22^Tononi and collaborators explicitly state this as a possibility through different versions of the integrated information theory: “Thus, the theory predicts that a brain where no neurons were activated, but were kept ready to respond in a differentiated manner to different perturbations, would be conscious (perhaps that nothing was going on)” (Tononi, 2004, p. 19) “Inactive elements specify the shape of a quale. The assumption that neural elements that are active are broadcasting information often goes hand in hand with the corollary that inactive elements are essentially doing nothing, since they are not broadcasting anything. According to the [integrated information theory], this is not correct. In the general case, being ‘off’ is just as informative as being ‘on’.” (Balduzzi and Tononi, 2009, p. 14) “[The integrated information theory] predicts that, even if all the neurons in a main complex were inactive (or active at a low baseline rate), they would still generate consciousness as long as they are ready to respond to incoming spikes.” (Oizumi et al., 2014, p. 17) “That silent neurons can contribute to consciousness is because, in IIT, information is not in the message that is broadcast by an element, but in the form of the conceptual structure that is specified by a complex. Inactive elements of a complex specify a cause-effect repertoire (the probability of possible past and future states) just as much as active ones (think of the dog that did not bark in the famous Sherlock Holmes story).” (Tononi and Koch, 2015. p. 10)

^23^This lack of pressure to choose among theories of consciousness is reflected in the way that the science of consciousness developed: there has been a massive influx of NCC-theories since the mid 1990s, but there is still no paradigmatic theory. There are nearly as many NCC-theories around as people attending conferences, making NCC-theories more like toothbrushes than anything else: everybody has their own, and one hardly wants to use someone else's. Additionally, hardly any *experimenta crucis* are reported, not even speaking of attempts to reject a theory. Instead, certain theories are merely abandoned: Lamme seems to have abandoned his own in favor of Tononi's, but we do not know why; Tononi changed the way to calculate Φ from article to article, but we do not know exactly how these patches are motivated. This way of progressing is suspicous, and old-fashioned inter-hypothetic comparison is to be preferred.

^24^The mapping of neural tokens correlating onto phenomenal tokens may be projective, surjective, injective, or bijective.

^25^That is: For the mapping of neural types onto phenomenal types, we only allow bijective mappings.

^26^For example, the phenomenal stability of the perceived world during wakefulness can hardly be maintained by neural activation alone. In dreams, one experiences a fleeting and changing scenery. A constantly constraining external force may be necessary for us to have any experience of stability.

## References

[B1] Aleksander I. Gamez D. (2011). Informational theories of consciousness: a review and extension. Adv. Exp. Med. Biol. 718, 139–147. 10.1007/978-1-4614-0164-3_12 21744216

[B2] Aru J. Bachmann T. Singer W. Melloni L. (2012). Distilling the neural correlates of consciousness. Neurosci. Biobehav. Rev. 36, 737–746. 10.1016/j.neubiorev.2011.12.003 22192881

[B3] Balduzzi D. Tononi G. (2009). Qualia: the geometry of integrated information. PLoS Comput. Biol. 5:e1000462. 10.1371/journal.pcbi.1000462 19680424 PMC2713405

[B4] Barrett A. B. Seth A. K. (2011). Practical measures of integrated information for time-series data. PLoS Comput. Biol. 7:e1001052. 10.1371/journal.pcbi.1001052 21283779 PMC3024259

[B5] Bayne T. (2010). The Unity of Consciousness. Oxford: Oxford University Press.

[B6] Bayne T. Hohwy J. (2013). Consciousness: theoretical approaches, in Neuroimaging of Consciousness, ed Cavanna A. E. (Berlin: Springer-Verlag), 23–35.

[B7] Block N. (1998). How to find the neural correlate of consciousness, in Royal Institute of Philosophy Supplement, eds Hameroff S. R. Kaszniak A. W. Scott A. C. (Cambridge, MA: MIT Press), 23–34.

[B8] Block N. (2005). Two neural correlates of consciousness. Trends Cogn. Sci. 9, 46–52. 10.1016/j.tics.2004.12.006 15668096

[B9] Borgstein J. Grootendorst C. (2002). Half a brain. Lancett 359, 473. 10.1016/S0140-6736(02)07676-6 11853792

[B10] Brentano F. (1874/1995). Psychology from an Empirical Standpoint. London: Routledge.

[B11] Chalmers D. J. (1996). The Conscious Mind: In Search of a Fundamental Theory. Oxford: Oxford University Press.

[B12] Chalmers D. J. (2000). What is a neural correlate of consciousness?, in Neural Correlates of Consciousness: Empirical and Conceptual Questions, ed Metzinger T. (Cambridge, MA: MIT Press), 17–39.

[B13] Corthout E. Chi-Hung B. U. Hallett J. M. Cowey A. (2000). Suppression of vision by transcranial magnetic stimulation: a third mechanism. NeuroReport 11, 2345–2349. 10.1097/00001756-200008030-00003 10943683

[B14] Crick F. (1995). The Astonishing Hypothesis: The Scientific Search For The Soul. New York, NY: Scribner.

[B15] Crick F. Koch C. (1990). Towards a neurobiological theory of consciousness. Semin. Neurosci. 2, 263–275.

[B16] Crick F. Koch C. (1995). Are we aware of neural activity in primary visual cortex? Nature 375, 121–123. 7753166 10.1038/375121a0

[B17] Crick F. Koch C. (1998). Consciousness and neuroscience. Cereb. Cortex 8, 97–107. 9542889 10.1093/cercor/8.2.97

[B18] Crick F. Koch C. (2003). A framework for consciousness. Nat. Neurosci. 6, 119–126. 10.1038/nn0203-119 12555104

[B19] Edlund J. A. Chaumont N. Hintze A. Koch C. Tononi G. Adami C. (2011). Integrated information increases with fitness in the evolution of animats. PLoS Comput. Biol. 7:e1002236. 10.1371/journal.pcbi.1002236 22028639 PMC3197648

[B20] Enns J. T. Di Lollo V. (2000). What's new in visual masking? Trends Cogn. Sci. 4, 345–352. 10.1016/s1364-6613(00)01520-5 10962616

[B21] Gamez D. (2008). Progress in machine consciousness. Conscious. Cogn. 17, 881–910. 10.1016/j.concog.2007.04.005 17572107

[B22] Gieryn T. F. (1983). Boundary-work and the demarcation of science from non-science: strains and interests in professional ideologies of scientists. Am. Sociol. Rev. 48, 781–795.

[B23] Griffith J. S. (1967). A View of the Brain. Oxford: Clarendon Press.

[B24] Heidegger M. (1986). Sein und Zeit. Tübingen: Niemeyer.

[B25] Hohwy J. (2007). The search for neural correlates of consciousness. Philos. Compass 2, 461–474. 10.1111/j.1747-9991.2007.00086.x

[B26] Hohwy J. (2009). The neural correlates of consciousness: new experimental approaches needed? Conscious. Cogn. 18, 428–438. 10.1016/j.concog.2009.02.006 19345590

[B27] Hohwy J. Frith C. D. (2004). The neural correlates of consciousness: room for improvement, but on the right track: comment. J. Conscious. Stud. 11, 45–51.

[B28] Holland O. Goodman R. (2003). Robots with internal models a route to machine consciousness? J. Conscious. Stud. 10, 77–109.

[B29] Joshi N. J. Tononi G. Koch C. (2013). The minimal complexity of adapting agents increases with fitness. PLoS Comput. Biol. 9:e1003111. 10.1371/journal.pcbi.1003111 23874168 PMC3708884

[B30] Koch C. (2004). The Quest for Consciousness: A Neurobiological Approach. Denver, CO: Roberts.

[B31] Koch C. (2012). Consciousness: Confessions of a Romantic Reductionist. Cambridge, MA: MIT Press.

[B32] Kuhn T. S. (1962/2012). The Structure of Scientific Revolutions. Chicago: University of Chicago Press; 50th anniversary edition edition.

[B33] Lamme V. A. F. (2004). Separate neural definitions of visual consciousness and visual attention: a case for phenomenal awareness. Neural Netw. 17, 861–872. 10.1016/j.neunet.2004.02.005 15288903

[B34] Lamme V. A. F. (2006). Towards a true neural stance on consciousness. Trends Cogn. Sci. 10, 494–501. 10.1016/j.tics.2006.09.001 16997611

[B35] Lamme V. A. F. Roelfsma P. R. (2000). The distinct modes of vision offered by feedforward and recurrent processing. Trends Neurosci. 23, 571–579. 10.1016/s0166-2236(00)01657-x 11074267

[B36] Lamme V. A. F. Zipser K. Spekreijse H. (1998). Figure-ground activity in primary visual cortex is suppressed by anesthesia. Proc. Natl. Acad. Sci. U.S.A. 95, 3263–3268. 9501251 10.1073/pnas.95.6.3263 PMC19730

[B37] Lamme V. A. F. Zipser K. Spekreijse H. (2002). Masking interrupts figure-ground signals in V1. J. Cogn. Neurosci. 14, 1044–1053. 10.1162/089892902320474490 12419127

[B38] Marshall H. R. (1901). Consciousness, self-consciousness and the self. Mind 10, 98–113. 26973491

[B39] Metzinger T. (2004). Being No One. Cambridge, MA: MIT Press.

[B40] Metzinger T. (2013). Two principles for robot ethics, in Robotik und Gesetzgebung, eds Holgendorf E. Günther J.-P. (Baden-Baden: Nomos), 263–302.

[B41] Mitchell W. (1933). The Place of Minds in the World. London: MacMillan & Co.

[B42] Montague R. (1960). On the nature of certain philosophical entites. Monist 53, 159–194.

[B43] Mormann F. Koch C. (2007). Neural correlates of consciousness. Scholarpedia 2:1740. 10.4249/scholarpedia.174026301908

[B44] Moutoussis K. Zeki S. (2002). The relationship between cortical activation and perception investigated with invisible stimuli. Proc. Natl. Acad. Sci. U.S.A. 99, 9527–9532. 10.1073/pnas.142305699 12089336 PMC123174

[B45] Murphy T. H. Corbett D. (2009). Plasticity during stroke recovery: from synapse to behaviour. Nat. Rev. Neurosci. 10, 861–872. 10.1038/nrn2735 19888284

[B46] Oizumi M. Albantakis L. Tononi G. (2014). From the phenomenology to the mechanisms of consciousness: integrated information theory 3.0. PLoS Comput. Biol. 10:e1003588. 10.1371/journal.pcbi.1003588 24811198 PMC4014402

[B47] Reggia J. (2013). The rise of machine consciousness: studying consciousness with computational models. Neural Netw. 44, 112–131. 10.1016/j.neunet.2013.03.011 23597599

[B48] Rose J. D. (2002). The neurobehavioral nature of fishes and the question of awareness and pain. Rev. Fish. Sci. 10, 1–38. 10.1080/20026491051668

[B49] Seth A. K. (2011). Causal density and integrated information as measures of conscious level. Philos. Trans. R. Soc. 208, 3748–3767. 10.1098/rsta.2011.0079 21893526

[B50] Seth A. K. Izhikevich E. Reeke G. N. Edelman G. M. (2006). Theories and measures of consciousness: an extended framework. Proc. Natl. Acad. Sci. U.S.A. 103, 10799–10804. 10.1073/pnas.0604347103 16818879 PMC1487169

[B51] Singer W. (2016). The ongoing search for the neuronal correlate of consciousness, in *Open Mind* eds Metzinger T. Windt J. M.(Cambridge, MA: MIT Press).

[B52] Stein D. G. Hoffman S. W. (2003). Concepts of CNS plasticity in the context of brain damage and repair. J. Head Trauma Rehabil. 18, 317–341. 10.1097/00001199-200307000-00004 16222128

[B53] Supèr H. Spekreijse H. Lamme V. A. F. (2001). Two distinct modes of sensory processing observed in monkey primary visual cortex (V1). Nat. Neurosci. 4, 304–310. 10.1038/85170 11224548

[B54] Tononi G. (2004). An information integration theory of consciousness. BMC Neurosci. 5:42. 10.1186/1471-2202-5-42 15522121 PMC543470

[B55] Tononi G. (2008). Consciousness as integrated information: a provisional manifesto. Biol. Bull. 215, 216–242. 10.2307/25470707 19098144

[B56] Tononi G. (2012a). Integrated information theory of consciousness: an updated account. Arch. Ital. Biol. 150, 56–90. 10.4449/aib.v149i5.1388 23165867

[B57] Tononi G. (2012b). PHI: A Voyage fromthe Brain tothe Soul. New York, NY: Pantheon Books.

[B58] Tononi G. Koch C. (2008). The neural correlates of consciousness: an update. Ann. N.Y. Acad. Sci. 1124, 239–261. 10.1196/annals.1440.004 18400934

[B59] Tononi G. Koch C. (2015). Consciousness: here, there and everywhere? *Philos. Trans. R. Soc. Lond. B Biol. Sci*. 370:20140167. 10.1098/rstb.2014.0167 25823865 PMC4387509

[B60] Weiss A. (1917). Relation between functional and behavior psychology. Psychol. Rev. 24, 353–368.

[B61] Wieloch T. Nikolich K. (2006). Mechanisms of neural plasticity following brain injury. Curr. Opin. Neurobiol. 16, 258–264. 10.1016/j.conb.2006.05.011 16713245

[B62] Wittenburg G. F. (2010). Experience, cortical remapping, and recovery in brain disease. Neurobiol. Dis. 37, 252–258. 10.1016/j.nbd.2009.09.007 19770044 PMC2818208

